# The role of the GABA system in amphetamine-type stimulant use disorders

**DOI:** 10.3389/fncel.2015.00162

**Published:** 2015-05-05

**Authors:** Dongliang Jiao, Yao liu, Xiaohong Li, Jinggen liu, Min Zhao

**Affiliations:** ^1^Shanghai Mental Health Center, Shanghai Jiao Tong University School of MedicineShanghai, China; ^2^Department of Neurochemistry, NY State Institute for Basic Research in Developmental DisabilitiesNew York, NY, USA; ^3^State Key Laboratory of Drug Research, Shanghai Institute of Materia Medica, Chinese Academy of SciencesShanghai, China

**Keywords:** GABA system, amphetamine-type stimulants use disorders, psychotic, cognitive dysfunctions, drug abuse

## Abstract

Abuse of amphetamine-type stimulants (ATS) has become a global public health problem. ATS causes severe neurotoxicity, which could lead to addiction and could induce psychotic disorders or cognitive dysfunctions. However, until now, there has been a lack of effective medicines for treating ATS-related problems. Findings from recent studies indicate that in addition to the traditional dopamine-ergic system, the GABA (gamma-aminobutyric acid)-ergic system plays an important role in ATS abuse. However, the exact mechanisms of the GABA-ergic system in amphetamine-type stimulant use disorders are not fully understood. This review discusses the role of the GABA-ergic system in ATS use disorders, including ATS induced psychotic disorders and cognitive dysfunctions. We conclude that the GABA-ergic system are importantly involved in the development of ATS use disorders through multiple pathways, and that therapies or medicines that target specific members of the GABA-ergic system may be novel effective interventions for the treatment of ATS use disorders.

## Introduction

Abuse of amphetamine-type stimulants (ATS), including amphetamine, methamphetamine (METH), 3,4-methylenedioxymethamphetamine (MDMA), and 3,4-methylenedioxyamphetamine (MDA), is a global public health problem. According to the United Nations World Drug Report, approximately 0.3–1.3% of the world's population suffers from ATS abuse problems (Burns, 2014). The use of ATS causes serious social problems, including violence (Pluddemann et al., 2010), criminal behavior (Cartier et al., 2006), and an increased prevalence of AIDs (Halkitis et al., 2001; Colfax and Guzman, 2006).

ATS comprise a class of psychoactive substances, and long-term use of ATS results in addiction (Cretzmeyer et al., 2003; Cruickshank and Dyer, 2009). However, large dose or long-term use of ATS may also result in psychiatric disorders or cognitive deficits (Batki and Harris, 2004; Curran et al., 2004). Psychotic symptoms include hallucinations and delusions (Bramness et al., 2012), which are similar to the clinical features of schizophrenia. According to the reports, the prevalence of psychosis in methamphetamine abusers ranges between 10 and 60% (Farrell et al., 2002; McKetin et al., 2006; Mahoney et al., 2008). Various animal behaviors, such as increased activation and hallucinogenesis in non-human primates (Castner and Goldman-Rakic, 1999), and increased locomotion activity, stereotyped behavior, deficits in prepulse inhibition and latent inhibition, or behavior sensitization in rodents (Robinson and Becker, 1986; Tenn et al., 2003; Featherstone et al., 2007; Chen et al., 2010; Macedo et al., 2012), resemble human psychiatric disorders. The clinical manifestations of ATS-induced cognitive dysfunction include (Scott et al., 2007; Jacobs et al., 2008) response inhibition and deficits in learning, memory, attention, and decision making, social cognition, executive functions, and working memory. Research in animal models demonstrates that ATS could cause impairment to cognitive functioning in animals (Belcher et al., 2005; Camarasa et al., 2010; Herring et al., 2010).

The major mechanism of ATS addiction is traditionally though to be increased activity of the mesolimbic dopamine system. However, until now, there has been a lack of effective medicines for treating ATS-related problems. Indeed, emerging evidence that will be discussed in this paper suggests that a GABA-ergic dysfunction may also result in ATS use disorders, including ATS-induced psychiatric disorders and cognitive dysfunction. We hope this review will help to elucidate the mechanisms underlying ATS use disorders as well as aid in the development of novel interventions.

This review focuses on the role of the GABA-ergic system in ATS use disorders. In the following manuscript, we first discuss the impacts of ATS on the GABA system, including GABA receptors, transporters, and so on. We further discuss addiction-related brain regions in ATS use disorders, such the prefrontal cortex, striatum, and the involvement of GABA system in the dysfunctions of these neural circuits. In addition, we also document the genetic evidence on the relationship between GABA system and ATS use disorders, and review some clinical applications of GABA system medicines to treat ATS use disorders. Thus, this review summarizes the involvement of GABA-ergic system in ATS use disorders, providing us with a new aspect on research on ATS use disorders.

## ATS change the functions of the GABA system

GABA, which is the main inhibitory neurotransmitter in the central nervous system (CNS), works with excitatory neurotransmitters to maintain the normal functions of the CNS. The GABA system, including GABA receptors, GABA transporters (GAT), and glutamate decarboxylase (GAD), is widely distributed in the CNS and is related to ATS use disorders.

### GABA receptors and ATS use disorder

GABA receptors include ionotropic receptors (GABA_A_ and GABA_C_) and metabotropic receptors (GABA_B_). The ionotropic GABA receptors are ligand-gated ion channels, which mediate fast inhibitory synaptic transmission in neurons, whereas the metabotropic GABA_B_ receptors belong to the superfamily of G-protein coupled receptors (GPCRs) which are responsible for the neuromodulation of GABA (Benarroch, 2012). Of these GABA receptors, GABA_A_, and GABA_B_ are the most well-studied receptors in ATS use disorders.

#### ATS influence the normal functions of GABA_A_ receptors

The ionotropic GABA_A_ receptors are the major inhibitory neurotransmitter receptors that mediate fast synaptic transmission inhibitory effects. GABA_A_ receptors are composed of five protein subunits that belong to different subunit classes, including subunits α1-6, β1-3, γ1-3, δ, ε, θ1-3, π, and ρ1-3 (Simon et al., 2004). The GABA_A_ α1β2γ2 receptor subtype combination is the most abundant subtype in most brain areas (McKernan and Whiting, 1996). Importantly, GABAergic signaling in dopaminergic brain areas, like the Ventral Tegmental Area (VTA) and the substantia nigra compacta, is also mainly mediated by the GABA_A_ α1β2γ2 receptor subtypes (Petri et al., 2002).

Recent research has shown that the use of ATS would influence the normal functions of GABA_A_ receptors. One study (Hondebrink et al., 2013) using a two-electrode voltage-clamp technique showed that administration of ATS in *Xenopus* oocytes expressing human GABA_A_ α1β2γ2 receptor subunits directly inhibited a GABA_A_ receptor-evoked electric current, possibly via competitive binding to one of the GABA binding sites, which represented decreased potential intensity. A similar study (Hondebrink et al., 2011) displayed an inhibitory effect by ATS on GABA_A_ receptor-induced inhibitory potential in the nervous system. If present *in vivo*, this decreased inhibitory GABA-ergic input on, for example, dopaminergic neurons, may resulted in enhanced drug-induced DA release and drug addiction. In support of this finding, animal studies have demonstrated that pretreatment with clonazepam (a GABA_A_ receptor agonist) prevented the acquisition of behavioral sensitization of methamphetamine in rats (Ito et al., 2000). Alternatively, antagonism of GABA_A_receptors aggravates ATS-induced disorders. Intra- prefrontal cortex (PFC) injection of a GABA_A_ receptor antagonist, dicentrine, enhanced amphetamine-induced psychomotor activation (Enomoto et al., 2011). The mechanism could be that the activation of GABA_A_ receptor decreases dopaminergic neurotransmission, which could be increased by inhibition of GABA_A_ receptor, suggesting that GABA_A_receptors may play an inhibitory role in ATS use disorders.

#### ATS influence the normal functions of GABA_B_ receptors

The GABA_B_ receptors are G-protein-coupled receptors with seven transmembrane domains. The two subunits of a GABA_B_receptor are GABA_B1_ and GABA_B2_. The GABA_B_ receptors are located on the pre- and post-synaptic sites. Activation of the presynaptic GABA_B_ receptors inhibits calcium ion flux from extracellular to intracellular sites by blocking calcium channels and thus inhibits transmitter release. Activation of the post-synaptic GABA_B_ receptors, however, activates the potassium channels and increases the intracellular potassium concentration, leading to neuron hyperpolarization and producing a slow inhibitory post-synaptic current (IPSC).

Recent research has shown that the use of ATS depressed the normal functions of the GABA_B_ receptors-GIRK (G-protein-gated inwardly rectifying potassium, GIRK/Kir3) channels. Padgett et al. (2012) showed that a single *in vivo* injection of METH depressed GABA_B_ receptors-GIRK channels in VTA GABA neurons. Thus, it enhanced VTA GABA neuron firing, which may augment GABA transmission in VTA. The VTA GABA neurons provide a local source of GABA for controlling the firing of VTA DA neurons. These neuro- adaptations in the GABA neurons are predicted to increase the GABA-mediated inhibition of VTA DA neurons. However, repeated psychostimulant administration leads to increased firing rates of VTA DA neurons and development of ATS addiction. Thus, the increase of GABA-mediated inhibition of VTA DA neurons, which is efficient at first, may suggest that short-term use of ATS would enhance the compensatory enhancement of GABA, whereas large doses and long-term use would eventually lead to depression in the GABA_B_ receptor signaling in DA neurons. Few research has examined the effects of methamphetamine on GABA_B_ receptors in DA neurons; however, the following evidences indicate that methamphetamine causes impairment to the GABA_B_ receptors in DA neurons.

Some studies have suggested that GABA_B_ receptor agonists may be useful for decreasing amphetamine-induced dopamine release and treating ATS use disorders. One study (Balla et al., 2009) showed that injection of GABA_B_ receptor agonists, SKF97541 or baclofen, into the PFC significantly reduced the basal dopamine level and decreased amphetamine-induced dopamine release in rats. The GABA_B_ receptor agonist baclofen could block the development of ATS- induced behavior sensitization (Bartoletti et al., 2005) and attenuate ATS self-administration in rats (Brebner et al., 2005). Baclofen and GABA_B_ positive allosteric modulators, GS39783 and CGP7930, which specifically activate the GABA_B_ receptors, inhibited the expression of methamphetamine-induced conditioned place preference (Halbout et al., 2011; Voigt et al., 2011). These findings indicate that the action of GABA_B_ receptor agonists may be a target for the treatment of ATS use disorders.

### Impact of ATS on other GABA system members (GAT, GAD, and glutamate)

The GABA transporter (GAT), another important member of the GABA system, is located at the synapses of the GABA-ergic neurons; GAT is responsible for GABA reuptake and thus maintains the basal GABA level in the extracellular space. GAT is also important for the regulation of synaptic transmissions of the GABA-ergic neurons. Four distinct transporters for the GABA are GAT-1, GAT-2, GAT-3, and BGT-1(Borden et al., 1995). Animal experiments have shown that when mice were treated with ATS, the immune activity of striatal GAT-1, a predominant GABA transporter, was enhanced, suggesting the possibility of an increased reuptake of GABA, which may be the main explanation for the decreased GABA level in the extracellular space (Izco et al., 2010).

GAD is a specific GABA synthetase and is positively correlated with the GABA levels in various brain regions. Two isoenzymes determined by different molecular weights are GAD65 and GAD67. In a rat behavioral sensitization mode (Zhang et al., 2006), methamphetamine was reported to decrease the GAD67 protein level in the NAc, suggesting an inhibitory effect on the GABA-ergic neurotransmitter caused by ATS. This inhibition is related to ATS-induced locomotor sensitization. This study also found that the GAD67 protein level increased in the striatum, which is consistent with the findings of another study (Pereira et al., 2008), which showed that amphetamine induced an elevation of the GABA levels in the striatum. Some opposing phenomena show that methamphetamine significantly decreased GAD67 mRNA and protein levels or the GABA level in the striatum (Jayanthi et al., 2004; Pereira et al., 2012). One possible explanation is that short-term or low-dose methamphetamine use (30 mg/kg, for 1 day) (Pereira et al., 2008) induced compensatory enhancement of the striatal GABA level, whereas long-term or high-dose methamphetamine use (40 mg/kg, for 7 days) (Jayanthi et al., 2004) induced apoptosis of the GABA-ergic neurons and decreased the level of GAD in the striatum of rats. The second explanation is the following difference in animal species; mice appear to be more susceptible to ATS than rats. The identical dosage of ATS (30 mg/kg) induced a compensatory enhancement of the GABA level in the striatum of rats (Pereira et al., 2008) and decreased the GABA level in C57BL/6 adult mice (Pereira et al., 2012).

GABA is derived from glutamate under the decarboxylation action of GAD, via glutamine-glutamate-GABA circulation; thus, changes in the glutamate level could affect the GABA content as well. Studies have found that ATS use could change the glutamate levels in the brain. Animal experiments have demonstrated that a single dose of ATS (30 mg/kg) in mice led to decreased levels of glutamine, glutamate, and GABA in the corpus striatum and down-regulated the glutamine/glutamate and GABA/glutamate ratios, suggesting a circulatory disturbance of the glutamine-glutamate-GABA circuit (Pereira et al., 2012). Bu et al. (2013) treated rats with 2.5 mg/kg of ATS twice a day for over 7 days and detected reduced GABA, glutamate, and glutamine levels in the PFC. Using magnetic resonance spectroscopy (MRS) in ATS addicts, Sailasuta et al. (2010) found abnormal glutamine-glutamate circulation in neuroglia cells. The reduction of the glutamate level was hypothesized to be due to over-excitation of the glutamate system induced by ATS, and an excessive need for glutamate was produced. The decreased GABA level was caused by an insufficiency of glutamate, the synthetic ingredient of GABA. Additionally, ATS could promote GABA metabolism into succinic acid semialdehyde, for which an increased level was found after the ATS treatment (Bu et al., 2013).

**Figure 1:**
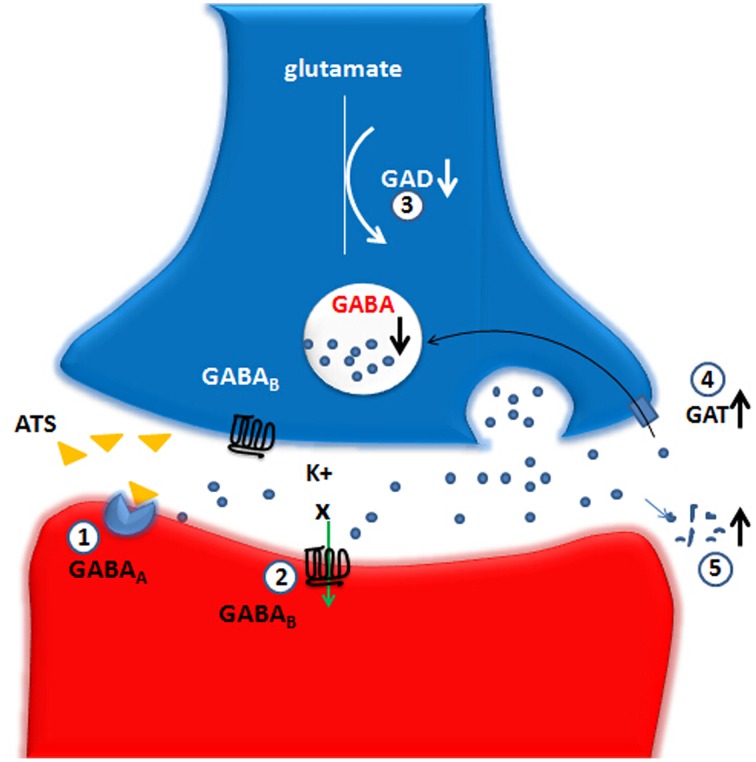
**Large doses and long-term use of ATS lead to an decrease on GABA-ergic system function**. The up and down arrows represent the decreased or increased level or function and the X marks represent the decreased function. GABA, gamma-aminobutyric acid; GAD, glutamic acid decarboxylase; GAT, GABA transporters; GABA_A_, GABA_A_ receptor; GABA_B_, GABA_B_ receptor. (1) GABA_A_ receptor: ATS directly inhibit a GABA_A_ receptor-evoked electric current, possibly via competitive binding to one of the GABA binding sites. (2) GABA_B_ receptor: ATS depress the normal functions of the GABA_B_ receptors-GIRK channels. (3) GAD: ATS decrease the GAD67 protein level in the NAc, suggesting an inhibitory effect on the biosynthesis of GABA-ergic neurotransmitter caused by ATS. (4) GAT: ATS increase the expression of striatal GAT-1, which could be a possible explanation for the reduction of extracellular GABA expression. (5) ATS could promote GABA metabolism into succinic acid semialdehyde.

The existing evidence showed that ATS can change the functions of multiple members of the GABA-ergic system. Although short-term use of ATS may enhance the compensatory enhancement of GABA, large doses and long-term use will lead to insufficient GABA synthesis and increased decomposition, thus decreasing GAD and GABA levels. ATS can reduce GABA_A_ and GABA_B_receptor functioning and increase the function of GAT. These changes by ATS eventually lead to a decrease in GABA-ergic system function (see Figure 1).

## ATS abuse impairs normal functions of GABA system in related regions

Abnormal activation of the dopamine systems in the nigrostriatal, mesolimbic, or mesocortical pathways have been regarded as the three classic pathways involved in drug addiction. Because the GABA system plays an important role in ATS addiction as well, the following section focuses on changes in the GABA system in these three classic pathways, including the ventral tegmental area (VTA), prefrontal cortex, and striatum in ATS use disorders.

### ATS disrupt the inhibitory function of GABA system in VTA

In the last decade, a large number of studies, at the cellular and animal behavioral levels, have shown that, under the stimulation effect of addictive drugs, projections from the VTA dopamine neurons, particularly the enhanced dopamine projection from VTA to NAc, are the main neural mechanisms underlying substance dependence (Luscher and Ungless, 2006; Schultz, 2006).

Additionally, ATS abuse is correlated with changes in the VTA-NAc pathway. Acute application of ATS increases dopamine release from the VTA to the NAc through the mesolimbic and mesocortical pathways (Kankaanpaa et al., 1998; Zhang et al., 2001). Over-activation of the NAc dopamine neurons produces euphoria and connects this emotion with conditional signals, thus constituting the neural basis of the induction of drug craving and drug-seeking behaviors (Haber and Knutson, 2010). Repeated or long-term use of ATS causes sustained adaptive changes in the mesolimbic circuit neurons, namely neural plasticity, which ultimately leads to a stable drug addictive state (Koob and Le Moal, 2001; Luscher and Malenka, 2011).

More than 30% of the neurons in the VTA are GABA-ergic neurons (Creed et al., 2014). There are two types of GABA-ergic neurons, as follows: GABA-ergic interneurons, which produce an inhibitory effect on local VTA dopamine neurons; and GABA-ergic projection neurons, which depress their projected brain regions. Some reports (Tan et al., 2012; van Zessen et al., 2012; Bocklisch et al., 2013) have demonstrated that when GABA-ergic neurons in the VTA are activated, the activation of dopaminergic neurons, locally and in its projected regions, are strongly inhibited. Alternatively, inhibition of the VTA GABA-ergic neurons causes an enhanced activation of dopaminergic neurons locally and in its projected regions. Many studies have shown that the GABA system plays an important role in the ATS-induced over-activation of the NAc dopamine neurons. One study (Giorgetti et al., 2002) showed that after 5 days of injection with 5 mg/kg ATS and 3 days of withdrawal, microinjection of the GABA_B_ receptor antagonist into the VTA in rats caused significant increases in the glutamate and dopamine levels compared with those in the non-ATS treated control group. The results indicated that under the effect of ATS, the brain would mobilize the GABA inhibitory effect in the VTA to maintain normal dopamine and glutamate levels in a self-protective manner. This imbalanced inhibitory effect might be a possible explanation for ATS use disorder, and this result was supported by another study (Bankson and Yamamoto, 2004; Padgett et al., 2012). All of these studies showed that protecting or enhancing the GABA inhibitory effect on dopamine and glutamate neurons in the VTA might provide a strategy for treating ATS addiction.

### ATS disturb the inhibitory function of the GABA system in PFC

The prefrontal cortex is unique to the evolution of primates. The human prefrontal cortex is involved in many complicated cognitive processes, such as logical thinking, working memory, decision making, and organization of behavior (Fuster, 2000; Brown and Bowman, 2002; Kasanetz et al., 2013). As part of the mesolimbic circuit, the PFC plays a very important role in drug abuse and addiction (Roberts et al., 1998; Daglish et al., 2003). Injury to the PFC could cause impairment of judgment, planning, and the capacity for decision making, the so-called “executive dysfunction syndrome” (Roberts et al., 1998). Recent research (Baler and Volkow, 2006; Kasanetz et al., 2013) hypothesized that an important explanation for addiction is impairment to the PFC, which results in executive dysfunction and leads to uncontrolled drug-taking behavior. As a result, the PFC dysfunction by ATS is responsible for ATS-induced psychosis and cognitive dysfunction and is closely related to ATS addiction.

In the primate neocortex, the GABAergic neurons constitute approximately 25–30% of the neuronal population (Jones, 1993). They are interneurons, and they maintain the normal functions of the PFC pyramidal cells though their inhibitory effects on the dopaminergic neurotransmitter (projecting from the VTA) and glutamatergic neurotransmitters (projecting from the thalamus) (Jones, 1993; Gabernet et al., 2005).

**Figure 2:**
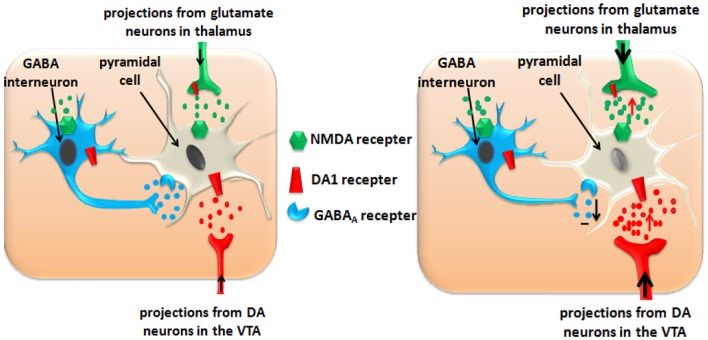
**ATS disturb the inhibitory function of GABA interneurons in PFC and impairs the function of PFC pyramidal cells. **Left panel**:** Normal state in the PFC. **Right panel**: ATS increase the glutamate and dopamine levels in the PFC, and exacerbate these effects by disturbing the inhibitory function of the GABA-ergic interneurons. Over-activation or long-term activation of the glutamatergic and dopaminergic neurons by ATS could produce excitatory neurotoxicity and cause pyramidal cell apoptosis.

Acute use of ATS increases the glutamate level in the PFC (Stephans and Yamamoto, 1994; Qi et al., 2009), and moderate activation of the glutamatergic neurons plays an important role in synaptic plasticity, which is closely related to cognitive learning (Luo et al., 2002; Abraham and Williams, 2003). Over-activation or long-term activation of the glutamatergic neurons by ATS could produce excitatory neurotoxicity and cause cell apoptosis. Apoptosis of the PFC pyramidal cells induces functional disorders of the PFC, and, finally, results in addiction, psychosis, and cognitive dysfunction (Deng et al., 2001; Kuczenski et al., 2007). Apoptosis of the PFC GABA interneurons reduces their inhibitory effects on the dopaminergic neurotransmitters and glutamatergic neurotransmitters (see Figure 2).

Additionally, acute use of ATS facilitates the projection of dopamine from the VTA to the PFC though the mesolimbic pathway (Haber and Knutson, 2010). The stimulation of the D1-like dopamine receptors located on the glutamatergic terminals induced enhancement of the glutamate release (Licata and Pierce, 2003), so dopamine further increased the excitatory neurotoxicity of glutamate. Repeated amphetamine treatment leads to potentiated interactions between DA and glutamate transmission (Giorgetti et al., 2001).

The PFC GABA-ergic interneurons suppress over-activation of the dopaminergic and glutamatergic neurons, and ATS might exacerbate these effects by disturbing the inhibitory function of the GABA-ergic neurons. As reported by Brummelte's group (Brummelte et al., 2007), early treatments with methamphetamine triggered changes in the post-natal maturation of the prefrontal cortical GABA-ergic neurons in Mongolian gerbils and led to a decreased inhibitory regulatory effect by the GABA-ergic interneurons. In another experiment (Enomoto et al., 2011), researchers used a GABA_A_ receptor antagonist, bicuculline, to block the GABA_A_ receptors in the PFC of rats and found a significant increase of ATS-induced locomotion in adult rats. This finding may be due to the uncontrolled over-activation of dopaminergic and glutamatergic neurons, which causes impairment to the PFC function and is closely related to addictive memories.

Microinjections of the GABA_B_ receptor agonists, baclofen, or SKF-97541, into the PFC of rats, significantly reduced the local extracellular dopamine basal level, as well as the ATS-induced dopamine release (Balla et al., 2009). Baclofen improved ATS-induced cognitive dysfunction, suggesting a relationship between the GABA system and PFC function (Arai et al., 2009). It could be hypothesized that improving the function of the GABA system would have a protective effect in ATS-induced PFC nerve injury, although there is no direct evidence from human studies.

### ATS abuse disrupts functions of the striatum and its related neural circuit via GABA system

#### ATS abuse impairs strital structure and functions

Accumulating data have confirmed that the striatum plays an important role in ATS use disorder. An MRI study (Chang et al., 2005) on ATS-dependent patients demonstrated that long-term use of amphetamine led to enlargements of the caudate nucleus, pallidum, and putamen of the basal nuclei; this enlargement maybe a local inflammatory reaction caused by amphetamines. Other sub-cortex regions, such as the thalamus, midbrain, cerebellum, and callosum, were unaffected. In people with a large accumulation of amphetamines or mental symptoms induced by ATS, the striatum becomes even smaller. This phenomenon suggests that long-term use of amphetamines may ultimately result in apoptosis of striatal cells and reduced striatum volume. In fetuses with long term prenatal methamphetamine exposure, the putamens, and pallidums were smaller than those in methamphetamine-free fetuses (Chang et al., 2004), suggesting the influence of ATS on the growth and development of children. Animal studies demonstrated that a large dose of methamphetamine induced neuronal apoptosis of the striatum neurons, which could be an important explanation for the shrinkage of the striatum (Jayanthi et al., 2004; Zhu et al., 2006).

#### Relationship between GABA system and the striatal related neural circuit (see Figure 3: top panel)

Approximately 77% of the striatal neurons are GABA-ergic projection neurons (Graveland et al., 1985) that project to different brain regions. One type of GABA-ergic neuron projects to the substantia nigra pars reticulata (SNr)/globus pallidus interna (GPi), which is the initial portion of the direct excitability pathway; D1 receptors are expressed on the surface of this type of GABA-ergic neuron. The other type of GABA-ergic neuron projects to the globus pallidus externa (GPe). The inhibitory GABA-ergic neurons project from the GPe to the subthalamic nucleus (STN). The glutamatergic neurons project from the STN to the SNr/GPi, which is referred to as the indirect pathway; these neurons are characterized by the surface expression of D2 receptors. The direct and indirect pathways converge in the SNr/GPi, the GABA-ergic neurons project from the SNr to the thalamus, and the glutamatergic neurons project from the thalamus to the cortex. Activation of the direct pathway excites the brain cortex, whereas excitement of the indirect pathway inhibits the activation of the cortex (Altar and Hauser, 1987; Haber and Knutson, 2010). The striatum receives projections from the dopaminergic neurons in the meso-substantia nigra pars compacta (Balleine et al., 2007; Nicola, 2007), and projections from the PFC glutamatergic neurons (Alexander and Crutcher, 1990; Haber and Knutson, 2010). These regions and pathways form the nigrostriatal and corticostriatal circuits, which may play an important role in ATS abuse.

**Figure 3:**
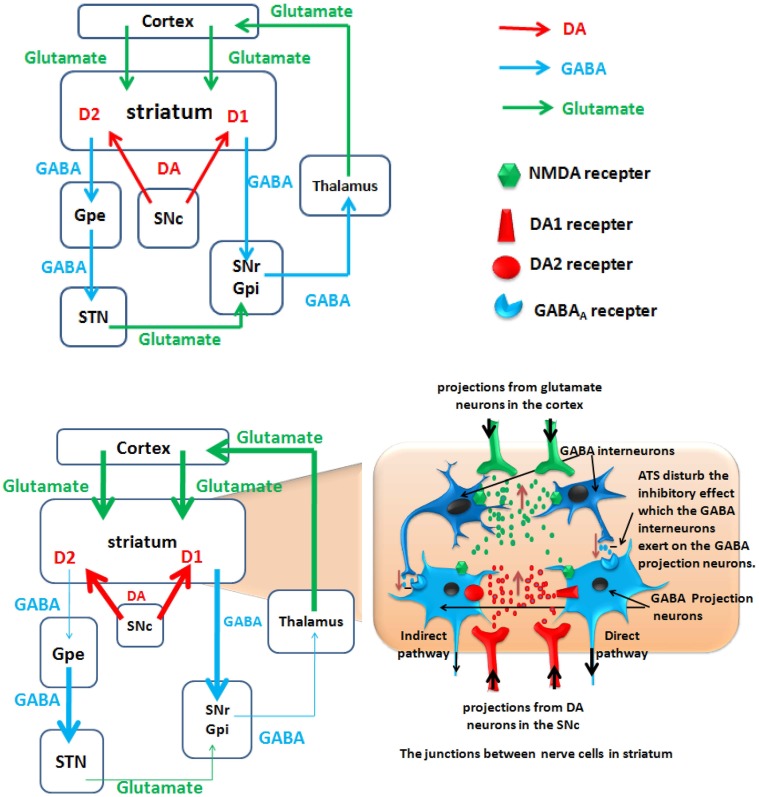
**A hypothetical model of polysynaptic effects causing ATS use disorders**. The degrees of enhancement and reduction are represented by the thickness of the lines. D2, dopamine D2 receptor; D1, dopamine D1 receptor; GPe, external globus pallidus; GPi, internal globus pallidus; STN, subthalamic nucleus; SNc, substantianigra pars compacta; SNr, substantianigra pars reticulate. **Top panel**: Normal state: Organization of intrinsic connections within the basal ganglia. The basal ganglia output is determined by the balance between the direct pathway and the indirect pathway. **Bottom panel**: ATS increase the glutamate and dopamine level in the striatum, and ATS disturb the inhibitory effect which the GABA interneurons exert on the GABA projection neurons. Under the action of dopamine, the D1 and D2 receptors cooperate to activate the direct pathway and to inhibit the indirect pathway. Through the activated direct pathway, the derepression release of glutamate projected from the thalamus to cortex ultimately increase glutamate release from the cortex to the striatum.

Interneurons within the striatum inhibit the GABA-ergic projection neurons (Rymar et al., 2004), and the neurons that express parvalbumin (PV) are the GABA-ergic interneurons. Parvalbumin-positive interneurons are the main type of interneurons that are identified as the junctions between afferent and efferent nerves (Kawaguchi et al., 1995; Plotkin et al., 2005). Compared with the GABA-ergic projection neurons, the GABA-ergic interneurons receive information from the cortex more easily and readily. After a quick response, all of the types of GABA-ergic projection neurons cause fast and strong inhibitory effects (Do et al., 2012). One study reported that the stimulation projected from the cortex activated GABA-ergic interneurons in the striatum, which inhibited the functions of the GABA-ergic efferent nerves, whereas the GABA_A_ receptor antagonist picrotoxin blocked this inhibitory effect between the interneurons and efferent nerves (Mallet et al., 2005). From these studies, we conclude that the GABA-ergic interneurons in the striatum adjust the function of the nigrostriatal and corticostriatal circuits.

#### Influence of ATS use disorders on GABA system in the striatum-related neural circuit (see Figure 3: bottom panel)

Acute administration of ATS enhances the striatal projections from the mesolimbic dopamine system (Ujike, 2002; Woodward et al., 2011) and the PFC glutamate system (Stephans and Yamamoto, 1994). The activated striatal neurons respond to the information transported from the PFC and produce changes in synaptic plasticity. Increased dopamine release in the striatum activates the GABA-ergic projection neurons that express D1 receptors and activates the direct pathway. The D2 receptor-expressing GABA-ergic projection neurons are suppressed (Surmeier et al., 2007), inhibiting the indirect pathway. Under the action of dopamine, the D1 and D2 receptors cooperate to activate the direct pathway and to inhibit the indirect pathway. Through the direct pathway, GABA-ergic neurons projected from the striatum to the SNr/GPi are activated, and GABA-ergic neurons projection from the SNr/GPi to the thalamus are inhibited (Altar and Hauser, 1987; Mark et al., 2004), leading to the derepression of the release of glutamate from thalamus to cortex (Mark et al., 2004) and ultimately promoting increased release of the cortical glutamate (Timmerman and Westerink, 1997). The high cortical glutamate level results in increased projection to the striatum through the corticostriatal pathway, which promotes positive-feedback action (Stephans and Yamamoto, 1994; Bellomo et al., 1998).

ATS inhibit the functions of the striatal GABA-ergic interneurons (Centonze et al., 2002) and decrease the benzodiazepine receptor binding sites of the striatal GABA_A_ receptors (Yoo et al., 2010). Large-dose injections of methamphetamine (40 mg/kg) over seven continuous days could activate the apoptotic cascade of mouse striatal GABA-ergic neurons (Jayanthi et al., 2004). Zhu et al. (2006) reported that a large dose of methamphetamine (30–40 mg/kg) induced 45% apoptosis of the parvalbumin-positive neurons in the striatum. However, the somatostatin-positive neurons could tolerate ATS-induced apoptosis. These results demonstrated that because of the massive release of glutamate and dopamine in the striatum, GABA interneurons in the striatum are susceptible to ATS-induced apoptosis and the accompanying cytotoxic effects (Nash and Yamamoto, 1992). Apoptosis of GABA-ergic interneurons in the striatum leads to decreased inhibition of GABA-ergic projection neurons and increases the release of GABA in the SNr, which would inhibit tonic GABA in the thalamus, allowing glutamate signals to fire in the cortex and further activate cortical neurons. Finally, the effect of ATS would enhance the positive-feed back action.

This evidence shows that ATS enhances the projections from the cortex to the striatum and promotes dopamine projections in the substantia nigra-striatum pathway, blocking the inhibitory effects of the GABA-ergic interneurons and enhancing the glutamate projections from the cortex to the striatum, thus finally improving the glutamate functions in the striatum. Through this positive feedback effect, long-time glutamate transmission promotes changes in the neural synaptic plasticity and ultimately results in the stabilization of addictive behavior (Yin and Knowlton, 2006; Balleine et al., 2007).

Therefore, we can conclude that the activation of the inhibitory effects of GABA-ergic interneurons in the striatum may produce a series of biochemical and behavioral changes. Researchers (Zhou et al., 2004) showed that systemic administration of the GABA_B_ receptor agonist, baclofen, reduced the release of striatal dopamine and improved ATS-induced hyper-activity. Few studies have reported the mechanisms of involvement of the GABA system in ATS abuse. Exploration in this field would offer new insight into the mechanisms involved in ATS abuse and facilitate the development of new medications for the treatment of ATS addiction and ATS-induced psychosis.

The biochemical function of the GABA system in ATS abuse is through an inhibitory mechanism. The GABA systems in the VTA, PFC, and striatum are connected by the mesolimbic, mesocortic, and corticostriatal pathways, and they participate in the development of ATS use disorders including ATS-induced psychosis, as well as injury to cognitive functioning. This finding provides a new insight into the treatment of ATS use disorders.

## Genetic studies on GABA system and ATS use disorders

### GABA system genes are associated with ATS use disorders

In recent years, numerous research on twins or adoptees suggested that genetics play a key role in drug use disorders (Vanyukov and Tarter, 2000). The effect of genetic factors is found in 33–79% of ATS abuse cases (Bousman et al., 2009). At present, the associative genes involved in ATS addiction are predominantly monoamine neurotransmitter genes, particularly those that regulate dopamine functions and neuropeptides and their receptors (e.g., prodynorphin and opioid receptors). Additionally, there are reports on the GABA system genes that are associated with ATS use disorders. A case-control study (Nishiyama et al., 2005) showed that the GABA_A_γ2 subunit is associated with ATS addictive behaviors. Another study (Lin et al., 2003) analyzed the GABA_A_ subunit genes located on chromosome 5q33 in ATS addicts and found that the human GABA_A_α1 (rs2279020) and γ2 (rs4480617) genes were associated with ATS addiction. The GABA_A_γ2 gene was reported to be involved in schizophrenia as well (Zai et al., 2009). These results indicate that the GABA system genes may be associated with ATS use disorders. Addictive drug abuse is hypothesized to be a polygenic inherited disease, and the GABA system genes may work with genes in other systems to participate in drug use disorders.

Genetic experiments on rodents showed that ATS could affect the function of the GABA system. Using two mouse lines selectively bred for high methamphetamine-induced activity (HMACT) or low methamphetamine-induced activity (LMACT), Palmer et al. (2005) found an increase in the GABA_B_1 gene expression level in the NAc tissues in the HMACT group. This finding may reflect a compensatory response to excessive activity of the VTA-NAc circuit in the HMACT line or heightened expression of GABA_B_1 because of a low resting tone of the ventral tegmental area of the GABA-ergic system.

### Epigenetic changes may be involved in ATS use disorders

Accumulating data show that epigenetic changes such as miRNA changes are involved in drug addiction (Sartor et al., 2012). Most research focuses on traditional addictive drugs such as cocaine, nicotine, alcohol, or opioids, and few studies have focused on ATS addiction. Animal studies manifested an enhanced miRNA181 level following ATS treatment (Saba et al., 2012), and such up-regulation was hypothesized to be closely associated with the development of schizophrenia (Beveridge et al., 2008). One of the target genes of miRNA181 is the GABA_A_α1 subunit (Zhao et al., 2012), which suggests a relationship between the GABA_A_ receptor and ATS use disorders that is in accordance with the above findings. Therefore, miRNA181 may be involved in ATS use disorders through regulation of the GABA genes. Validated data are lacking, and further studies are needed.

### Experimental genetic techniques studies

Experimental genetic techniques are an important source of new knowledge about the interrelationship of genetic factors and behavioral outcomes in substance abuse. Studies using the molecular genetic technique, including gene silencing or knock-out methods confirmed the involvement of the GABA system in ATS use disorders. Adopting bacterial, artificial chromosome-driven and miRNA silencing technology, the Schmidt research group (Schmidt et al., 2014) generated a transgenic mouse line, in which glutamic acid decarboxylase 1 (GAD1) expression was suppressed in cholecystokinin (CCK) or neuropeptide Y (NPY)-expressing GABA-ergic interneurons. They measured the locomotor responses of these transgenic mice following a single dose of amphetamine at 3 mg/kg. Their results showed that the NPYGAD1TG mice (mice with GAD1-suppressing bacterial, artificial chromosome constructs in NPY+ interneurons) displayed a significantly greater increase of up to a 600% peak response to amphetamine compared with the littermate controls. In a separate experiment, the CCKGAD1TG mice (mice with GAD1-suppressing bacterial, artificial chromosome constructs in CCK+ interneurons) showed a approximately 50% reduction in the peak response. A possible explanation was that CCK+ and NPY+ GABA-ergic interneurons were distributed to different areas in the brain, which induced complex functional changes in the brain neural circuits and consequently led to different behavioral responses (Chronwall et al., 1985; Meziane et al., 1997). Elucidation of the exact mechanisms requires further studies.

Researchers (Reynolds et al., 2003) pretreated GABA_A_α1 knock-out mice with amphetamines and found that their stereotypical responses were enhanced whereas no changes were seen in the hyper-locomotor responses. Because the hyper-locomotorresponse is mainly correlated with nucleus accumbens (Pijnenburg and van Rossum, 1973; Kelly and Iversen, 1976) and the stereotypical response is related to the dorsal striatum (Joyce and Iversen, 1984; Staton and Solomon, 1984), the changes in the stereotypical responses in amphetamine-treated GABA_A_α1knock-out mice indicate that the dorsal striatal GABA_A_α1 subunit may play a more important role in ATS abuse.

## GABA system targeted medicine and its clinical applications in ATS use disorders (see Figure 4)

This evidence demonstrates that the GABA system is involved in ATS use disorders, and interventions focused on the GABA system may provide a possible novel treatment for ATS use disorders. Some studies have attempted to use GABA system medicines to treat ATS use disorders. Benzodiazepines, which are GABA_A_ receptor agonists, are frequently used in the clinic to treat psychiatric symptoms caused by ATS-induced central nervous system hyper-activation (Merckmanuals^1^). Pre-administration of benzodiazepines, such as alprazolam (0.5 mg), could attenuated the discriminative stimulus effects of D-amphetamine, and some of the self-reported drug effects (Rush et al., 2004). The use of another GABA_A_ receptor agonist, topiramate (100–200 mg/d), could protect ATS-induced memory impairment and reduce drug cravings (Johnson et al., 2007). The administration of the GABA_B_ receptor agonist, baclofen (20 mg/d), was reported to reduce the ATS dosage (Heinzerling et al., 2006). Additionally, the GAT inhibitor, vigabatrin (1–3 g/d), could reduce the dosage of ATS and has been shown to be safe in humans (Brodie et al., 2005). Although the above data demonstrate that medicines targeting the GABA system are potential treatments for ATS use disorders, there are some limitations.

**Figure 4:**
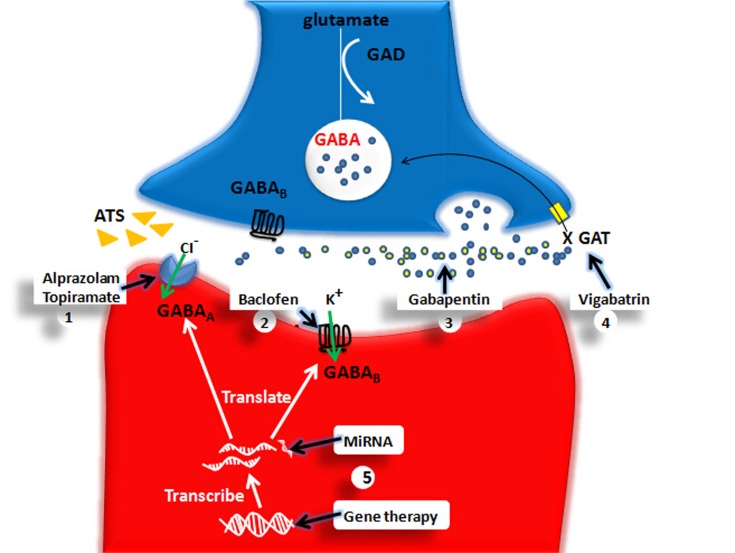
**The pharmacological and genetical therapeutical strategies for ATS use disorders**. The X marks represent the decreased function. GABA, gamma-aminobutyric acid; GAD, glutamic acid decarboxylase; GAT, GABA transporters; GABA_A_, GABA_A_ receptor; GABA_B_, GABA_B_ receptor. (1) Alprazolam and Topiramate: They enhance GABA_A_-mediated an influx of chloride ions further to promote rapid inhibitory neurotransmission. (2) Baclofen: Baclofen enhances GABA_B_-mediated an influx of potassium thus induces a slow inhibitory postsynaptic current. (3) Gabapentin: GABA analog. (4) Vigabatrin: As a GAT inhibitor, Vigabatrin decreases reuptake of GABA and increases GABA level in the extracellular space. (5) Gene Therapy and MiRNA may be ideal therapeutic choice for ATS use disorders in the future. For example, MiRNA181 may modify GABA_A_α1 gene expression at posttranscriptional level and gene therapy may modify GABA_A_α1, GABA_A_γ2 gene mutation.

First, some medications targeting the GABA system (e.g., benzodiazepines) are addictive themselves, which limits their clinical applications. Second, the curative effects of the currently used GABA system medicines could only partially inhibit the ATS effect, and their efficacy is minimally satisfactory. Therefore, more effective medicines are needed for ATS use disorders. Third, only a small amount of GABA system medicines are used in the treatment of ATS addiction in the clinic, and the available effective clinical data are not sufficient to establish safe guidelines for clinical applications. Fourth, gene mutation and miRNAs are involved in addiction-related behaviors. That therapies that target specific genes or miRNAs may be novel effective interventions for the treatment of ATS use disorders.

## Conclusion

The majority of previous studies on ATS use disorders focused on the meso-corticolimbic dopamine system. The role of the GABA system in ATS use disorders has received increasing attention in recent years. This review focused on the role of the GABA system and discussed the role of the GABA system in the three classic pathways, as well as genetic research and clinical applications for ATS use disorders. This review may provids a better understanding of mechanisms of the GABA-ergic system in ATS use disorders. We find that ATS can have effect on multiple members of the GABA-ergic system and eventually lead to a decrease in GABA-ergic system function. The complex relationships between GABA, dopamine and glutamic acid in ATS use disorder, as well as the resulting pathological and physiological changes, are manifested through the interaction between neural circuits. We conclude that the GABA-ergic system are importantly involved in the development of ATS use disorders through multiple pathways, and that therapies or medicines that target specific members of the GABA-ergic system may represent new avenues for the treatment of ATS use disorders.

Further studies are needed for a better understanding of the mechanism of the GABA system in ATS use disorders and the search for a better treatment.

A considerable part of the explanation for ATS use disorders attribute to the genetic effects. Researches on GABA system-related genetic studies are not as much as the other systems such as dopamine nowadays, which the future researches should focus on. Epigenetics researches such as miRNAs and their relationship with ATS use disorders are just started and less well-studied, but still there are evidence certifying that ATS abuse changes certain miRNAs expression. Future studies may reveal more complex and intriguing properties of miRNAs in ATS addiction. The molecular genetic technique of gene targeting to create mice with specific gene knockout mutations in the central nervous system should be further employed to gain insight into the molecular and cellular basis of ATS addiction. This helps to clarify the interaction between ATS use disorders and GABA system.

Although GABA system medicines are already applied clinically to treat ATS addiction associated symptoms, because of the sedative effect or addictive characteristic by themselves, their therapeutic effects are heavily discounted. Genetic studies demonstrate that the influence of ATS on GABA system may be more specific to GABA receptor subunits. For example, human genetic researches showed the GABA_A_α1, GABA_A_γ2 subunits were associated with ATS addictive behaviors, and animal genetic researches discovered the correlative relationship between GABA_A_α1, GABA_B_1 and ATS addiction. Therefore, discovering more targeted and effective medicines aiming at GABA receptor subunits maybe a new developmental direction in the future. In addition, in the practical treatments on opioids withdrawal, researchers found that drug combination was more effective, and would significantly reduced drug desire. Thus, we hope a similar drug combination strategy could be applied clinically and finally play a part in ATS use disorders.

## Author contributions

All authors have materially participated in the manuscript preparation. DJ wrote the first draft of the manuscript. YL provided critical revision of the manuscript for important intellectual content. All authors gave input to the manuscript text and approved the final version of the manuscript.

### Conflict of interest statement

The authors declare that the research was conducted in the absence of any commercial or financial relationships that could be construed as a potential conflict of interest.

## Footnotes

^1^http://www.merckmanuals.com/professional/special_subjects/drug_use_and_dependence/amphetamines.html
